# Digital quantification of the MMSE interlocking pentagon areas: a three-stage algorithm

**DOI:** 10.1038/s41598-024-59194-1

**Published:** 2024-04-19

**Authors:** Namhee Kim, Timothy Truty, S. Duke Han, Moonseong Heo, Aron S. Buchman, David A. Bennett, Shinya Tasaki

**Affiliations:** 1 Michael Reese Foundation Center for Health Equity Research, Rosalind Franklin University of Medicine and Science, 3333 Green Bay Road, North Chicago, IL 60064 USA; 2 Rush Alzheimer’s Disease Center, Rush University Medical Center, Chicago, IL 60612 USA; 3 Department of Family Medicine, University of Southern California, Los Angeles, CA 90089 USA; 4 Department of Neurology, University of Southern California, Los Angeles, CA 90089 USA; 5 Department of Psychology, University of Southern California, Los Angeles, CA 90089 USA; 6 School of Gerontology, University of Southern California, Los Angeles, CA 90089 USA; 7 Department of Public Health Sciences, Clemson University, Clemson, South Carolina 29634 USA

**Keywords:** Mini-mental state examination, Pentagon copying test, Aging, Perceptual speed, Computational biology and bioinformatics, Neuroscience, Medical research, Mathematics and computing

## Abstract

The Mini-Mental State Examination (MMSE) is a widely employed screening tool for the severity of cognitive impairment. Among the MMSE items, the pentagon copying test (PCT) requires participants to accurately replicate a sample of two interlocking pentagons. While the PCT is traditionally scored on a binary scale, there have been limited developments of granular scoring scale to assess task performance. In this paper, we present a novel three-stage algorithm, called Quantification of Interlocking Pentagons (QIP) which quantifies PCT performance by computing the areas of individual pentagons and their intersection areas, and a balance ratio between the areas of the two individual pentagons. The three stages of the QIP algorithm include: (1) detection of line segments, (2) unraveling of the interlocking pentagons, and (3) quantification of areas. A set of 497 PCTs from 84 participants including their baseline and follow-up PCTs from the Rush Memory and Aging Project was selected blinded about their cognitive and clinical status. Analysis of the quantified data revealed a significant inverse relationship between age and balance ratio (beta = − 0.49, *p* = 0.0033), indicating that older age was associated with a smaller balance ratio. In addition, balance ratio was associated with perceptual speed (r = 0.71, *p* = 0.0135), vascular risk factors (beta = − 3.96, *p* = 0.0269), and medical conditions (beta = − 2.78, *p* = 0.0389). The QIP algorithm can serve as a useful tool for enhancing the scoring of performance in the PCT.

## Introduction

The Mini-Mental State Examination (MMSE), introduced by Folstein et al.^1^. in 1975, is a widely used 30-point evaluation for screening delirium and cognitive impairment in clinical and research settings. One of the items in the MMSE is the pentagon copying test (PCT), which requires participants to replicate a sample of two interlocking pentagons on a paper. This subtest is used to measure visual-spatial construction abilities and provides psychomotor information about fine motor coordination, and attention to detail.

The traditional scoring of the PCT in the MMSE is binary; (correct = 1) if their drawing has ten angles and two pentagons that intersect. Several studies explored more detailed levels of qualitative scoring beyond the traditional binary scoring. One study further categorized the cases with a score of zero, based on traditional binary scoring, into five subclasses based on the degree of deviation from the interlocking pentagon sample used for testing^2^. Additionally, several studies proposed scoring systems that were not conditioned on the traditional binary scoring. For instance, one study proposed an 8-point scoring system using size of the figure, number of pentagons, fragmentation of the drawing, rotation, motor perseveration, and pull to stimulus^3^. Another study proposed a composite score, which was the sum of five domain scores based on the number of angles, distance/intersection between two pentagons, closure/opening of the image contour, rotation, and closing-in^4^. Furthermore, a study expanded scoring items to 15, including severity of distortion, breaks and corrections, balance of sizes between two pentagon, and total size^5^.

PCT scores were explored for their association with clinical outcomes and neuropsychological domains. Participants with Lewy Body Dementia (LBD) exhibited poorer performance compared to those with Alzheimer’s Dementia (AD)^4,6^ . Likewise, Participants with ischaemic vascular dementia (IVD) and Parkinson’s Disease (PD) showed poorer performance than those with AD, although there were few differences between IVD and PD^7^. Participants with Alzheimer's disease (AD) performed worse than the normal control group^2,8^. Additionally, PCT scores differentiated patients with schizophrenia from normal controls^5^. PCT scores showed an association with executive function assessed with word list generation, while their association with other cognitive domains such as semantic memory, declarative memory, and executive control was not established^7^.

To enhance efficiency and objectivity, automatic scoring approaches utilizing Deep Learning (DL) techniques, such as U-Net and convolutional neural networks, were proposed^9–11^. A study developed DL method for a mobile application, which employed a convolutional network known as U-net along with mobile sensor data^9^. Another study adopted a convolutional neural network utilizing an object detection model to generate an automatic traditional binary score^11^. While two preceding studies focused on training deep learning models to automate two well-established scoring systems^1,4^, a study devised a deep learning approach to explore an optimal scoring method for correlation with cognition^10^. Additionally, eight crucial drawing characteristics were identified through simulations using synthetic interlocking pentagon images including differences in sizes between two pentagons, overall pentagon size, and distance between the pentagons.

While various qualitative scorings that classify PCT performances into a range of deficits in visual-spatial construction abilities by visual inspection have been applied and proven effective, they also come with certain limitations. Qualitative scoring by rater’s visual inspection is subject to rater-to-rater variability and is available only in binary scale per item (score = 1 if a PCT meets a certain criteria). Recent advances in PCT scoring, equipped with DL techniques, have enhanced objectivity in classification of PCTs. However, there is still a demand for nuanced quantification for PCTs. For example, a balance ratio between two pentagons in continuous scale provides a more nuanced measure than a binary scoring^5^. This nuanced approach could allow more accurate and timely diagnosis of an individual’s cognitive status in both clinical and research settings.

In this study, among many qualitative features investigated for PCT, we focused three attributes based on the previous studies^5,10^that showed associations with cognitive and clinical outcomes: balance ratio in sizes between two pentagons, proportion of intersecting area, and total size of the PCT. We hypothesized that older adults, especially those with impaired visual-spatial construction skills due to increased medical conditions, vascular risk factors, cognitive aging, and various neurodegeneration, may encounter difficulty accurately reproducing the sample interlocking pentagons. This difficulty could be reflected in several ways, including a smaller total area, a reduced proportion of intersecting area, and an imbalance in the area between two pentagons.

**Figure 1:**
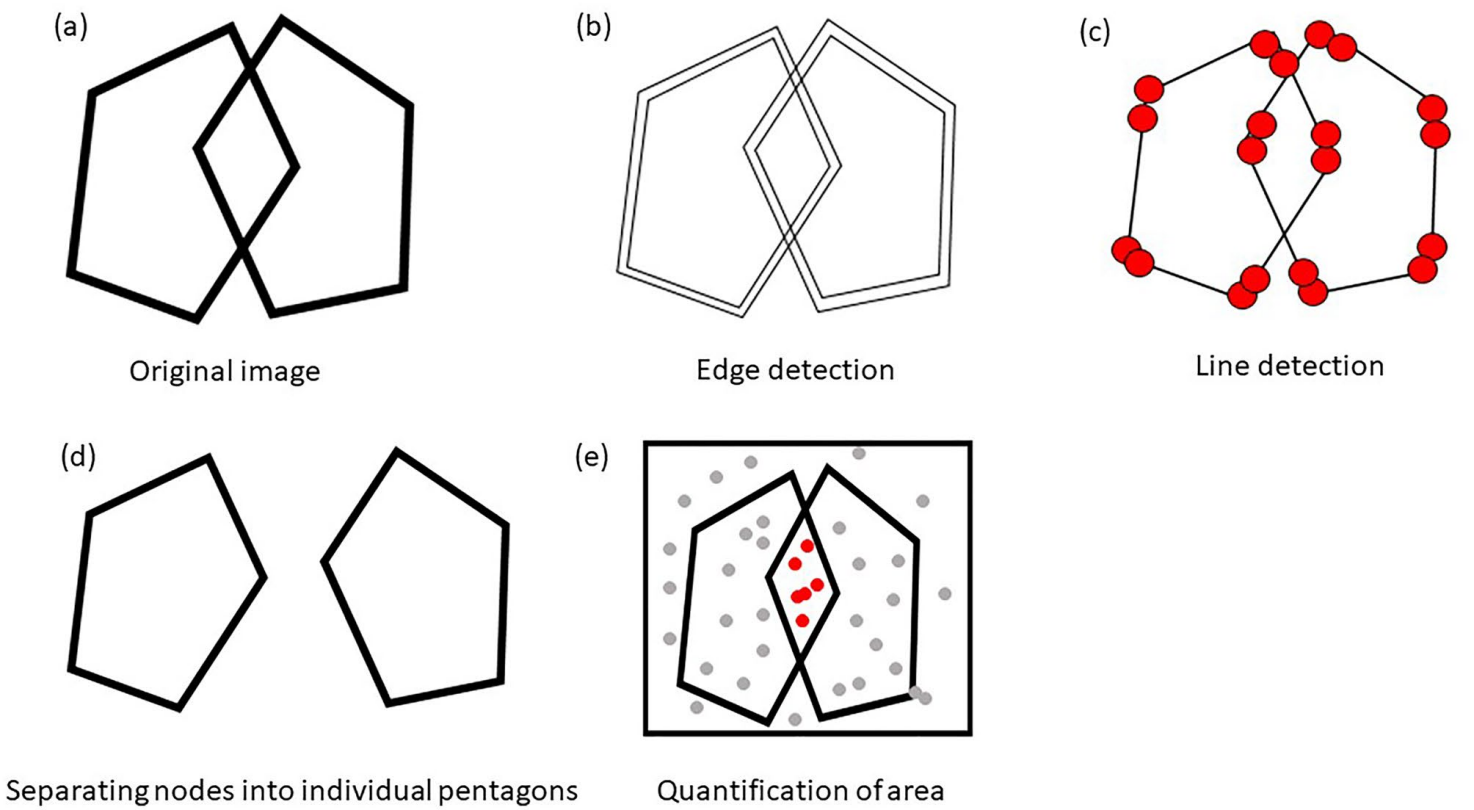
The diagram of the QIP algorithm. (**a**) Image of an interlocking pentagon, (**b**) Output of edge detection using the Canny edge detection algorithm, (**c**) Line segments detected through the Hough transformation, (**d**) Disentangled pentagons by clustering algorithms, and (**e**) Quantification of areas using the Monte Carlo integration.

We developed a three-stage algorithm called Quantification of Interlocking Pentagon (QIP) to quantify the three metrics mentioned above. A diagram of the QIP algorithm is provided in Fig. 1. We note that the proposed QIP algorithm can be applied to any two convex polygons besides pentagons, such as triangles, rectangles, or hexagons, whether or not the polygons intersect. These erratic drawings receive a score of zero according to the traditional binary scoring system. Comparing QIP metrics produced from pentagons to those from rectangles would not be meaningful because two cases represent different cognitive stages beyond what the algorithm can explain. Therefore, we included PCTs for this study that met the criteria for a "correct" condition (score = 1) based on the traditional binary scoring. The algorithm comprises line segment detection, unraveling of the interlocking pentagons, and quantification of relevant areas. We quantified 497 PCTs (all PCTs including both baseline and follow-up visits) from 84 participants randomly selected from the Rush Memory and Aging Project (MAP)^12^, an ongoing cohort study investigating aging and dementia, while cognitive status and MMSE scores were blinded for the selection. The three metrics from the QIP algorithm were associated with demographic variables, five cognitive domains, the composite scores, and medical conditions. We present the results of our study in Sect. 2, followed by a discussion in Sect. 3. We provide detailed information about the materials, methods and statistical analysis used in Sect. 4. Additionally, Supplementary Materials are included to offer descriptions of the QIP algorithm components.

## Results

The participants’ average age was 81.4 years (SD 5.8), with education 15 years on average (SD 2.7) at baseline. In the sample, there were 24 men (28.6%) and 83 non-Latino White (98.9%). Participants had follow-up visits on average 6.5 years (SD 3.4). At baseline, participants had an average of 1.4 medical conditions (SD 1.1), 0.9 vascular risk factors (SD 0.8), and 0.4 vascular diseases (SD 0.7). Demographic data of participants are described in Table 1.

**Table 1:** Baseline demographic distribution and total number of visits from all participants (*n* = 84).

Variable	Mean	SD or %	Min	25^th^	75^th^	Max
Age at baseline	81.4	5.8	68.1	77.2	85.6	97.8
Education	15.0	2.7	8.0	13.0	16.0	23.0
Male (*n*, %) Non-Latino White (*n*, %)	24 83	28.6 98.9	– –	– –	– –	– –
Number of total visits	6.5	3.4	1	4	9	13
N of medical conditions	1.4	1.1	0.0	1.0	2.0	4.0
N of vascular diseases	0.4	0.7	0.0	0.0	1.3	2.7
N of vascular risks	0.9	0.8	0.0	0.0	1.0	3.0
NCI (*n*, %)	55	65.5	–	–	–	–
MCI (*n*, %)	25	29.8	–	–	–	–
Dementia (*n*, %)	4	4.8	–	–	–	–

No Cognitive Impairment (NCI); Mild Cognitive Impairment (MCI).

**Figure 2:**
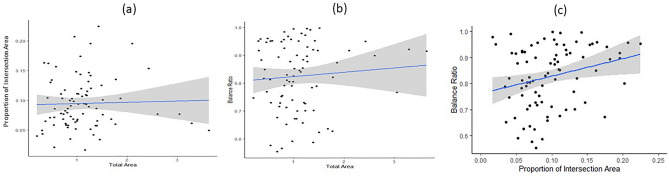
Association among three area measures from the QIP algorithm. Pairwise associations were examined among three quantified measures: total area, proportion of intersecting area, and balance ratio. The associations were demonstrated as follows: (**a**) the association between proportion of intersecting area and total area, (**b**) the association between balance ratio and total area, and (**c**) the association between balance ratio and proportion of intersecting area.

We examined the distribution of three metrics obtained from the QIP algorithm at baseline. On average, the proportion of intersection to the total area was 9.8% (SD 4.4%). The balance ratio, which represents the ratio of the smaller pentagon to the larger pentagon, was 82.2% (SD 12.2%). The ratio of the total area to the sample interlocking pentagon administered for PCT was 138% (SD 78%) (Table 2). As a reference, the sample interlocking pentagon displayed a proportion of intersection of 6.2% and a balance ratio of 99.0%. We observed a positive correlation between the balance ratio and the proportion of intersecting area (Spearman Correlation = 0.274, *p* = 0.0117), indicating that a higher balance ratio was associated with a greater proportion of intersection (Table 3 and Fig. 2).

**Table 2:** Summary of PCT quantification at baseline (n = 84).

	Mean	SD	Min	25%	75%	Max
Total area (%)	139	78	25	91	162	456
Prop. intersection (%)	9.8	4.4	1.7	6.5	12.7	22.5
Balance ratio (%)	82.2	12.2	55.4	71.9	93.5	99.9

Total area was divided by the total area of the sample interlocking pentagon administered for PCT.

**Table 3:** Spearman Correlation among three metrics of PCT at baseline (*n* = 84).

	Prop. intersection area	Balance ratio
Total area	0.0244 (*p* = 0.8536)	0.06637 (*p* = 0.5486)
Prop. intersection area	1.0	0.27387 (*p* = 0.0117)

**Figure 3:**
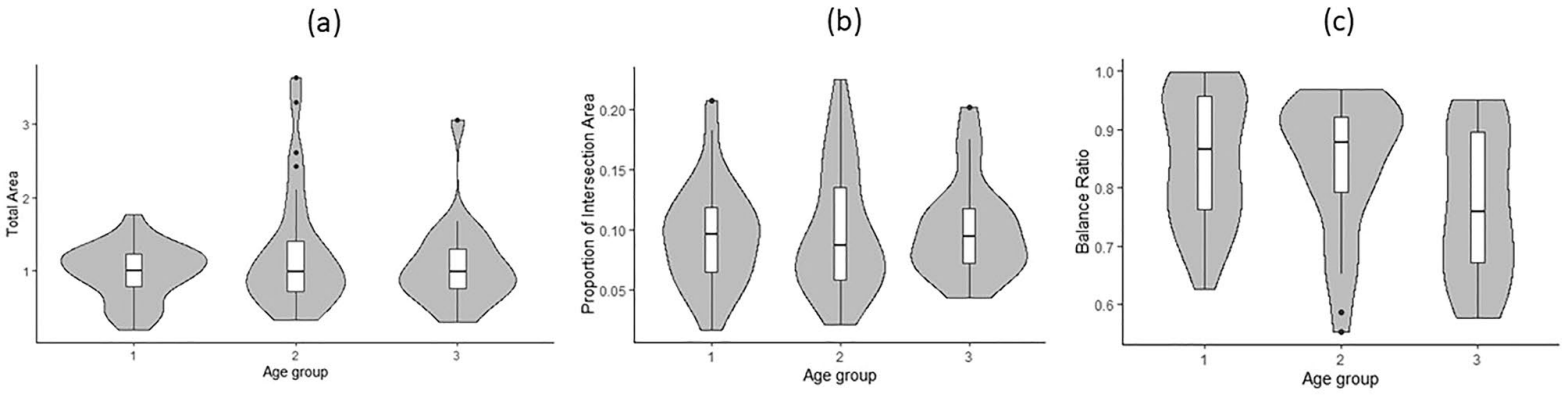
Distribution of three metrics from the QIP algorithm. The distribution of the three measures (total area, proportion of intersection, and balance ratio) was compared among three age groups at baseline, categorized by age tertiles (age < 79, 79 ≤ age < 82, age ≥ 82).

**Figure 4:**
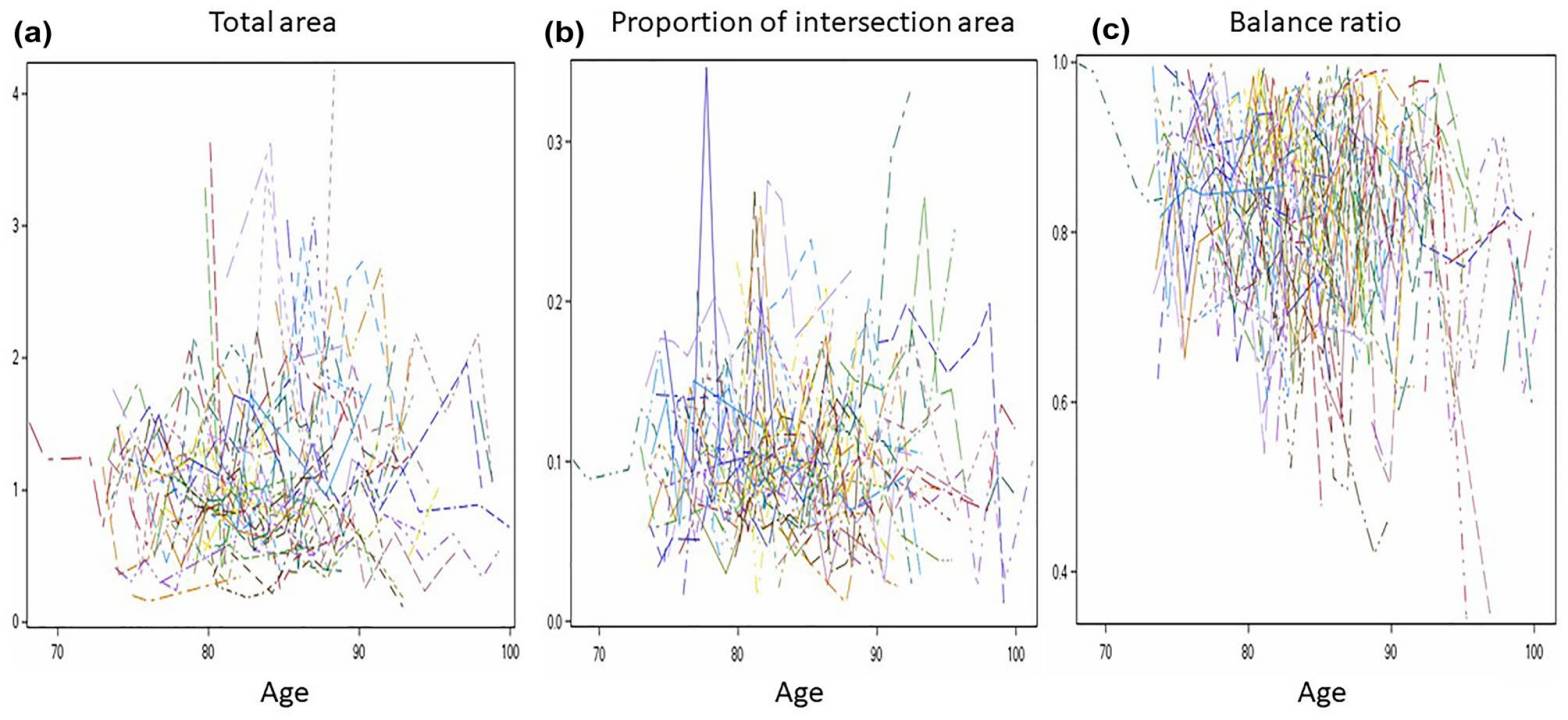
Longitudinal patterns of three metrics from the QIP algorithm. Spaghetti plots of the three area measures derived from the QIP algorithm were presented as follows: (**a**) Total area, (**b**) Proportion of intersection area, and (**c**) Balance ratio.

Figure 3 illustrates the distribution of the three measures (total area, proportion of intersection, and balance ratio) among three age groups at baseline based on tertiles (age < 79, 79 ≤ age < 82, age ≥ 82). Spaghetti plots depicting the longitudinal measures against age are presented in Fig. 4. Linear mixed-effects models were employed to analyze the relationship between each longitudinal measure (total area, proportion of intersection, and balance ratio) and age at baseline, sex, years of education, lag (in years) since enrollment of the study, and the interaction between lag and the three demographic measures with 497 PCT cases from 84 participants. We assessed the normality assumption of each QIP metric as an outcome of the mixed effects model, and determined that all three metrics were acceptable for normality assumption^13,14^. The results showed a significant inverse relationship between age and the balance ratio (beta = −0.49, *p* = 0.0033), indicating that a two-year increase in age at baseline was associated with approximately a 1% decrease in the balance ratio. Furthermore, the balance ratio exhibited a decline over the follow-up visits (beta = −5.44, *p* = 0.0394), suggesting an annual decrease of approximately 5.4%. Furthermore, we observed significant interaction effects between age and lag (beta = 0.07, *p* = 0.0372) as well as between sex and lag (beta = −1.06, *p* = 0.0104), indicating that the associations of balance ratio with age at baseline and sex change over the course of follow-up visits. Additionally, education was found to be associated with the proportion of intersecting area, with every 2-year increase in education being associated with a 1% decrease in the proportion of intersecting area (beta = −0.53, *p* = 0.0005). However, no significant relationship was found between the total area and any of the three demographic variables. A summary of these results can be found in Table 4.

**Table 4:** Linear mixed effects model with three longitudinal PCT measures.

	Effect	Estimate	SE	Pr >|t|
Total area (%)	Age at baseline	0.62	1.20	0.6070
	Sex	9.92	15.84	0.5318
	Education	−1.51	2.62	0.5643
	Lag	5.50	15.11	0.7170
	Age*Lag	−0.07	0.19	0.7115
	Sex*Lag	0.06	2.34	0.9789
	Education*Lag	−0.23	0.37	0.5320
Proportion of Intersection (%)	Age at baseline	−0.06	0.07	0.3524
	Sex	0.43	0.92	0.6413
	Education	−0.53	0.15	0.0005***
	Lag	0.18	0.99	0.8601
	Age*Lag	0.00	0.01	0.8753
	Sex*Lag	0.03	0.16	0.8706
	Education*Lag	0.01	0.02	0.5745
Balance Ratio (%)	Age at baseline	−0.49	0.17	0.0033**
	Sex	2.89	2.21	0.1909
	Education	−0.17	0.35	0.6265
	Lag	−5.44	2.56	0.0370*
	Age*Lag	0.07	0.03	0.0372*
	Sex*Lag	−1.06	0.41	0.0104*
	Education*Lag	0.07	0.06	0.2730

*− *p*-value < 0.05; ** *p* value < 0.01 *** *p* value < 0.001; No multiplicity correction was applied.

Finally, we examined association of the three QIP metrics with neuropsychology five cognitive domain scores and the composite scores and medical conditions. We found a significant correlation between the change in balance ratio and the change in perceptual speed, indicating that a slower decline in balance ratio was associated with a slower decline in perceptual speed over follow-up visits (r = 0.7040, *p* = 0.0135). These results are summarized in Table 5. At baseline, we found that an increase in medical conditions was associated with a 3% decrease in the balance ratio (*p* = 0.0389), while an increase in vascular risk factors was associated with a 4% decrease in the balance ratio (*p* = 0.0269). These results are summarized in Table 6.

**Table 5:** Correlation between change in each PCT measure and change in neuropsychological measure.

		Estimate	SE	Pr >|t|
Total area	Total MMSE	0.3765	0.3734	0.3133
	Global cognition	0.3928	0.2841	0.1668
	Episodic memory	0.3456	0.2785	0.2147
	Semantic memory	0.4162	0.2909	0.1524
	Working memory	0.2202	0.4222	0.6019
	Perceptual speed	0.3650	0.3052	0.2318
	Perceptual orientation	0.2480	0.3346	0.4587
Proportion of Intersection (%)	Total MMSE	−0.2035	0.2664	0.6550
	Global cognition	0.0002	0.0022	0.9999
	Episodic memory	0.3057	0.3642	0.4012
	Semantic memory	−0.2085	0.3777	0.5810
	Working memory	−0.0642	0.5226	0.9023
	Perceptual speed	−0.0738	0.3678	0.8411
	Perceptual orientation	0.4498	0.3562	0.2068
Balance Ratio (%)	Total MMSE	0.6492	0.3784	0.0863
	Global cognition	0.4557	0.4491	0.3102
	Episodic memory	0.4897	0.4952	0.3227
	Semantic memory	0.6834	0.5453	0.2102
	Working memory	0.2765	0.5750	0.6306
	Perceptual speed	0.7040	0.2851	0.0135*
	Perceptual orientation	−0.0884	0.4572	0.8467

*− *p*-value < 0.05; ** *p* value < 0.01 *** *p* value < 0.001; No multiplicity correction was applied.

**Table 6:** Association of three QIP metrics with medical conditions at baseline.

		Estimate	SE	*P*-value
Total Area	Number of medical conditions	9.03	7.87	0.2553
(%)	Number of vascular diseases	−0.03	14.53	0.9984
	Number of vascular risk factors	0.80	10.17	0.9379
	Cognitive impairment	26.04	16.27	0.1141
Proportion of	Number of medical conditions	−0.36	0.50	0.4733
Intersection (%)	Number of vascular diseases	−0.43	0.91	0.6407
	Number of vascular risk factors	0.72	0.66	0.2782
	Cognitive impairments	0.82	1.05	0.4380
Balance Ratio (%)	Number of medical conditions	−2.78	1.32	0.0389*
	Number of vascular diseases	0.04	2.50	0.9879
	Number of vascular risk factors	−3.96	1.75	0.0269*
	Cognitive impairment	−0.41	2.89	0.8895

*− *p*-value < 0.05; ** *p* value < 0.01 *** *p* value < 0.001; No multiplicity correction was applied.

Participant was classified as cognitively impaired (score = 1) if diagnosed with MCI or dementia, and as cognitively normal (score = 0) otherwise.

## Discussion

The QIP approach provides a more detailed quantification compared to the traditional binary scoring, which only assesses the presence of ten angles and the intersection of two pentagons. The QIP algorithm provides three metrics: total area, intersecting area, and balance ratio. We found that a lower balance ratio showed a significant association with older age at baseline, longer duration (in years) since enrollment of the study, lower perceptual speed, greater number of medical conditions, and vascular risk factors. We also found an association of proportion of intersecting area with education, indicating that a smaller proportion of intersecting area was associated with higher education. However, we found no association of total area with the demographic, cognitive, and medical conditions examined in this study.

While the association of balance ratio with the demographic, cognitive, and medical conditions was fairly straightforward and consistent with hypotheses, there may be more complicated implication with both total and intersecting area. For example, a very large intersecting or total area compared to the sample PCT may also indicate deficits in graphomotor skills in copying the sample PCT^3,7^, while it is well accepted that very small areas signify deficits^5^. This warrants further investigation.

The algorithm encountered difficulties when processing images of poor scan quality, high waviness, or instances of overshooting. In cases of low scan quality, the lines often blended with the background, causing Stage 1 to fail in detecting the lines. Drawings with high waviness or overshooting resulted in the inaccurate calculation of pentagon areas during Stage 3 of the QIP, owing to disparities between the original image and the reconstructed image based on the convex hull approach. It is worth noting that low scan quality could stem from ongoing neurodegeneration as studied in Tasaki et al.^10^ manifesting as reduced hand strength and muscle weakness of older adults. On the other hand, overshoots might be reflective of an individual's distinctive drawing style. For PCTs with low scan quality and high waviness, we classified them as failures of the algorithm. Meanwhile, to address overshoots, we introduced an extra manual step between Stage 2 and 3 for images exhibiting this trait. In this step, nodes forming overshoots were removed to rectify the issue.

While the QIP algorithm uses a digitized image from traditional PCT administration with paper and pencil as its input, recent advancements in digital technology applied to the graphomotor test have demonstrated the capability of quantifying performance to a greater extent. This has enabled a further detailed understanding of the cognitive process during the graphomotor test, such as thinking speed. Combining QIP with such a cutting-edge digital technology may further enhance nuanced quantification and understanding of participant’s cognitive status^16^.

This study has several limitations. Firstly, our developed QIP algorithm does not classify shapes in PCT to determine whether the polygons drawn were pentagons or not. Secondly, in this study, we applied the QIP algorithm to quantify PCTs that meet the requirements for the good condition based on the traditional binary scoring, excluding cases with a score of zero that indicate the severity of cognitive impairment. To achieve a more robust application, subsequent research should emphasize the integration of pre-existing DL methods with the QIP, enabling a comprehensive approach for both classifying and quantifying PCTs. Additionally, the computational complexity of Stage 2 of the QIP algorithm, which incorporates random permutation, warrants further improvements for enhanced efficiency. Lastly, we examined the associations between the three QIP metrics and cognitive and medical conditions with a small sample size (*n* = 84). Future studies should investigate with a larger sample size and consider including assessments of motor abilities of older adults.

## Materials and methods

### Ethical statement

This study adhered to the principles outlined in the Helsinki Declaration of 1964 and its subsequent amendments. All procedures involving human participants were approved by an Institutional Review Board of Rush University Medical Center, and informed consent was obtained from all participants before their inclusion in the study. Confidentiality and anonymity of participants were strictly maintained throughout the research procedure.

### Materials

#### Participants and their PCTs included for quantification

The MMSE has been administered for cognitive tests for all cohort studies at Rush Alzheimer’s Disease Center. Common eligibility criteria in both studies included: age > 65 years, absence of known dementia at the time of enrollment, and agreement to annual clinical evaluations. Of over 5000 participants tested with the MMSE at least once, we originally selected 90 participants from Memory Aging Project (MAP), an ongoing cohort study investigating aging and dementia, while blinding the cognitive status, MMSE scores and medical conditions. All 557 PCTs administered to the participants at baseline and in follow-up years were examined.

To validate the QIP algorithm, we included a subset of PCTs for quantification that met the condition for a score of one based on the traditional binary scoring which requires ten angles and two pentagons to intersect. Additionally, we note that the proposed QIP algorithm can be applied to any two convex polygons besides pentagons, such as triangles, rectangles, or hexagons, whether or not the polygons intersect. However, comparing QIP measures produced from pentagons (score = 1) to those from rectangles (score = 0) would not be meaningful because two cases represent different cognitive stages beyond what the QIP measures can explain. Therefore, we conditioned the inclusion of cases with a score of one based on the traditional binary scoring. Of 557 PCTs, 56 were scored zero and were therefore excluded. The excluded images exhibited arbitrary shapes, including single pentagons, two interlocking rectangles, no intersection between the two pentagons, or images with unintentional movements. Supplementary Fig. 1 presents examples of these excluded cases. A total of 501 PCTs with a score of one from 85 participants were included for quantification using the QIP algorithm. We evaluated the QIP algorithm’s performances with the original images manually. Among the 501 PCTs, the algorithm failed in quantification for 4 PCTs. These challenges were attributed to very low scan quality (*n* = 3), where lines were not distinguishable from the background that led to failure of detection of lines from the image at Stage 1 of the QIP, or highly wavy drawings that led to failure of accurate estimation of pentagon areas at Stage 3 of the QIP (*n* = 1). Consequently, a total of 497 PCTs from 84 participants were successfully quantified.

#### Neuropsychological and medical conditions

Participants underwent a comprehensive neuropsychological assessment. Episodic memory was measured using immediate and delayed story recall^17^, Logical Memory^18^, Word List Memory, Word List Recall, and Word List Recognition^19^. Semantic memory was measured using a 15-item version^19^ of the Boston Naming Test^20^, a verbal fluency test involving naming animals and vegetables/fruits in one-minute epochs^17,19^, and a 15-item word reading recognition test from the National Adult Reading Test^17^. Working memory was measured using Digit Span Forward and Backward^18^ and Digit Ordering^17^. Perceptual speed was measured using the oral form of the Symbol Digit Modalities Test^21^ and a modified version of Number Comparison^17^. Perceptual orientation was measured using a 15-item form of Judgment of Line Orientation^22^ and a 12-item form of Standard Progressive Matrices^24^. A global cognitive score was formed by averaging z scores of all tests. To create the composite, raw scores from the individual cognitive scores were converted to *z-*scores using the baseline mean and standard deviation of all participants enrolled in the parent cohorts. Each participant’s standardized *z*-scores were then averaged to yield a composite global cognition score, as previously described^25^. Cognitive test data were reviewed by a neuropsychologist to determine cognitive status at each visit. To provide a cognitive diagnosis, participants were evaluated by a clinician who used all cognitive and clinical data available. Dementia and its causes were diagnosed using the guidelines of the joint working group of the National Institute of Neurological and Communicative Disorders and Stroke and Alzheimer’s Disease and Related Disorders Association^23^. Diagnosis of MCI was given to individuals who had cognitive impairment but did not meet criteria for dementia.

Medical conditions are a self-reported composite measure of 7 medical conditions: hypertension, diabetes, heart disease, cancer, thyroid disease, head injury with loss of consciousness and stroke. A composite measure using the seven items was created by summing the number of conditions present (range: 0–7). Three vascular risk factors, smoking, diabetes and hypertension were obtained through self-report and a summary of vascular risk was created by summing the number of conditions present (range: 0–3). Vascular disease burden was computed using self-report questions for the four items: claudication, stroke, heart conditions, and congestive heart failure, where heart conditions include self-reported heart attack or coronary, coronary thrombosis, coronary occlusion, myocardial infarction. A cumulative score for vascular disease burden was created (range: 0–4).

### The QIP algorithm

The PCT was administered on paper, the paper was scanned, and then saved in the portable network graphics (png) format. The digital image was the input for the algorithm. Each digital image was first transformed into a binary image, where pixels are with zero for background and a positive constant for participant’ drawing. We, instead of using a massive number of individual nonzero pixels in each digital image for analysis, utilize line segments each of which is a collection of nonzero pixels.

The algorithm consists of three stages: (1) line segment detection from the image, (2) unraveling of two interlocking pentagons, and (3) quantification of the areas of interest. The first stage of detecting line segment is preceded by edge detection for which we applied the Canny edge detection^24^ that skeletonizes image. Once the edges are detected, the Hough transformation^25^ was applied to detect line segments, each of which is characterized by their starting and ending points in Euclidean space. In the second stage, to unravel two interlocking pentagons, we clustered the Hough line segments into individual pentagons based on connectivity matrix using the algorithm we developed. The third stage quantifies areas of individual pentagons using the Monte Carlo integration^26^. Flow of the algorithm is shown in Fig. 1. Details of the QIP algorithm were presented in Supplementary materials.

### Statistical analysis

We used univariate linear mixed effects models to assess change in each of the three QIP metrics (total area, proportion of intersection, balance ratio) over the follow-up years. The models included age at baseline, sex, education, lag (elapsed time at follow-up since baseline visit), and the interaction between lag and the three demographic variables as fixed effects. Additionally, subject-specific intercepts and slopes were included as random effects to account for variation between subjects.

To determine whether changes in QIP metrics over the follow-up years were associated with changes in cognitive scores (e.g., Is a slow decline in balance ratio associated with a slow decline in cognitive score?), we employed bivariate mixed effects models for each pair of QIP metrics and neuropsychological test scores (including total MMSE score, global cognition, episodic memory, semantic memory, working memory, perceptual speed, perceptual orientation) as the bivariate outcomes. These models also included age at baseline, sex, education, lag (elapsed time at follow-up since baseline visit), and the interaction between lag and the three demographic variables as fixed effects, as well as subject-specific intercepts and slopes for each bivariate outcome. The correlation of interest was estimated based on the covariance between two random slopes for the two outcomes^27^.

Additionally, each QIP metric was assessed for its association with each medical condition (including number of medical conditions, number of vascular diseases, number of vascular risk factors, and cognitive diagnosis), with age, sex, and education as covariates. All the statistical models were fitted using SAS 9.4.

### Supplementary Information

Supplementary Information 1.

Supplementary Information 2.

## Data Availability

The datasets used and/or analyzed during the current study available from the RADC Research Resource Sharing Hub (www.radc.rush.edu) under the terms of the data usage agreement. If you have any questions about the RADC Research Resource Sharing Hub, please contact RADC Data Sharing Coordinator Greg Klein at Gregory_Klein@rush.edu.
