# Supplementary material for: Digital quantification of the MMSE interlocking pentagon areas: a three-stage algorithm
Source: Sci Rep. 2024 Apr 19;14:9038. doi: 10.1038/s41598-024-59194-1 (PMC11031600; doi:10.1038/s41598-024-59194-1)
Supplement: Supplementary file 1 — Supplementary Information 1. [file 41598_2024_59194_MOESM1_ESM.pdf]

Supplementary Figures

Supplementary Figure 1

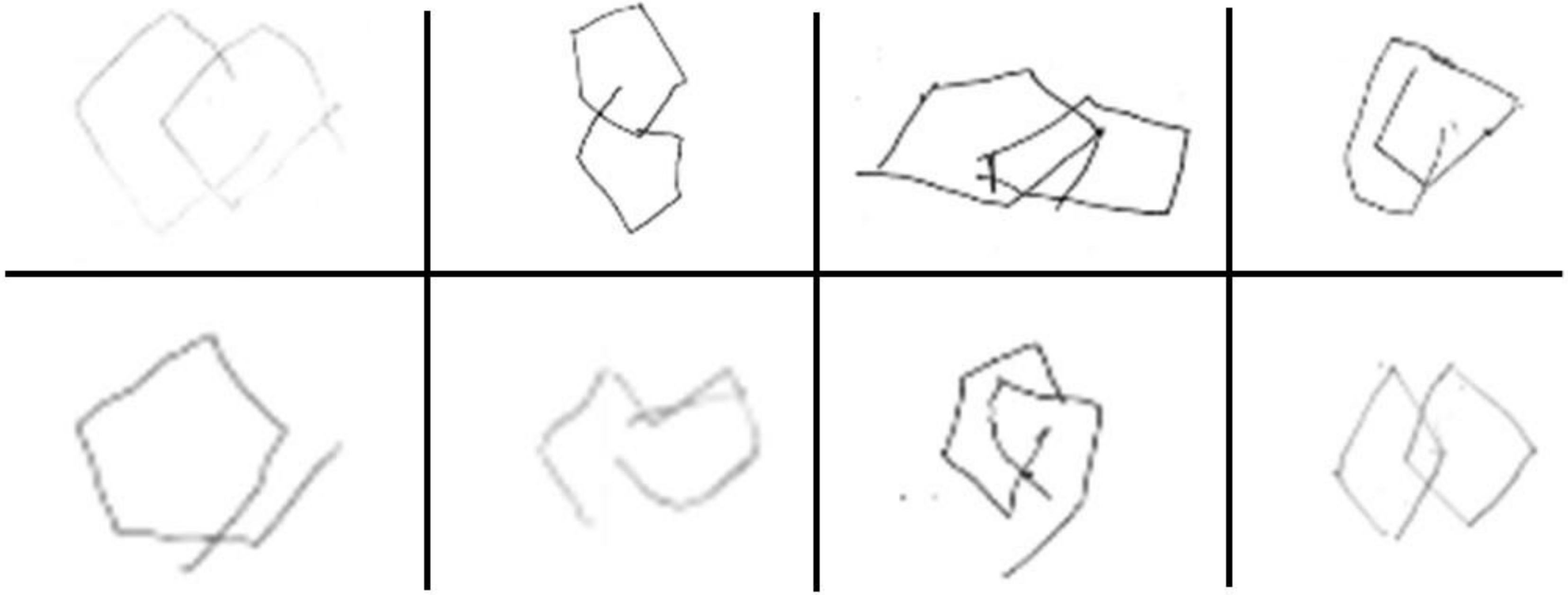

Supplementary Figure 1. Examples of PCTs that failed the binary scoring criteria. Eight PCT images received a score of zero based on the binary scoring method.

Supplementary Figure 2

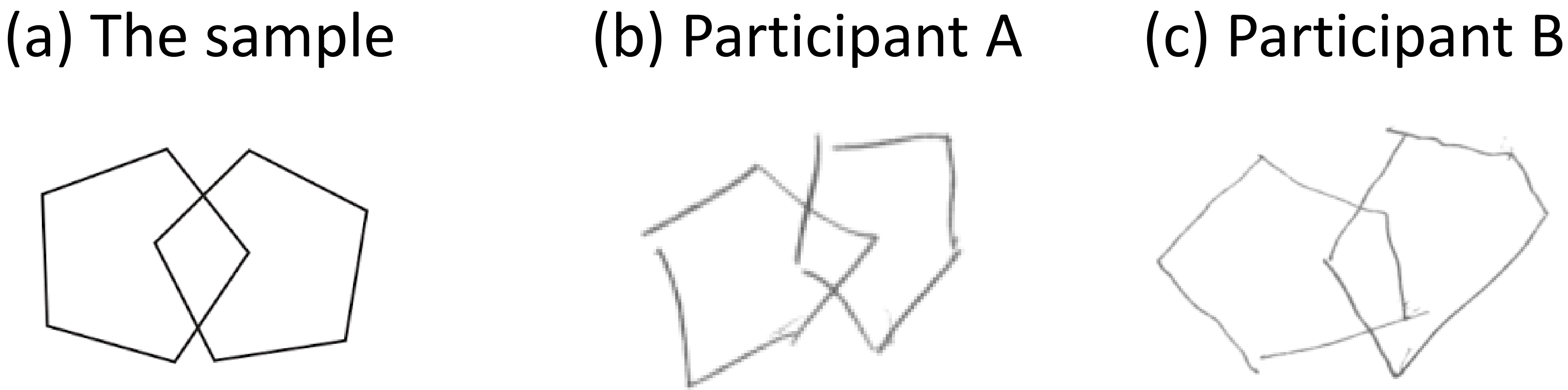

Supplementary Figure 2. Three PCT images for demonstration. The sample interlocking pentagon administered for PCT, participant A, and B. Participant A was a 79.2 years old man with 15 years of education, Participant B was a 91.6 years old man with 12 years of education.

Supplementary Figure 3

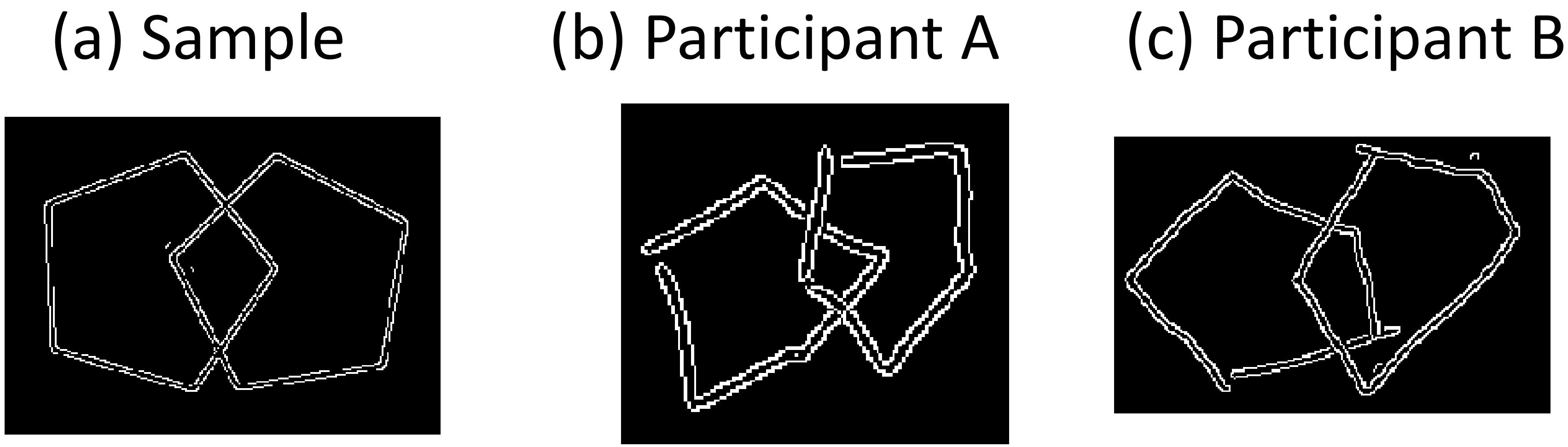

Supplementary Figure 3. The Canny Edge Detection. The Canny edge detection method was applied to delineate the edges of each original PCT image shown in Supplementary Figure 2.

Supplementary Figure 4

(a) Sample (b) Participant A (c) Participant B

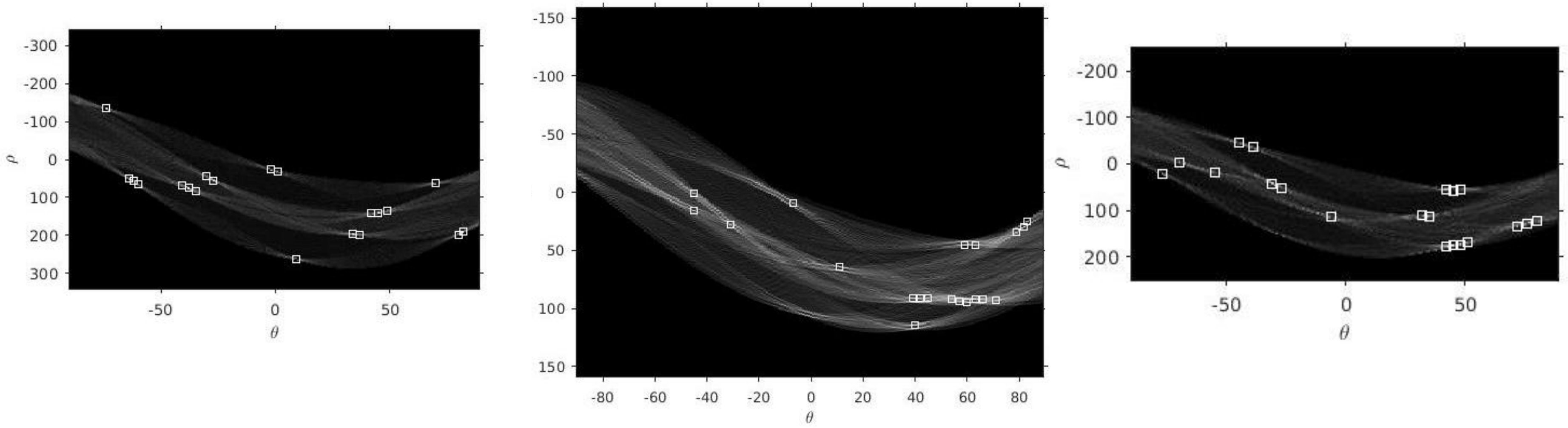

Supplementary Figure 4. The Hough transformation. The Hough transformation with three demonstrated examples are represented graphically, where the x-axis corresponds to the angle (θ) between the perpendicular line to each line segment and the positive x-axis, and the y-axis represents the distance between the perpendicular line and the point (0, 0).

Supplementary Figure 5

(a) Sample (b) Participant A (c) Participant B

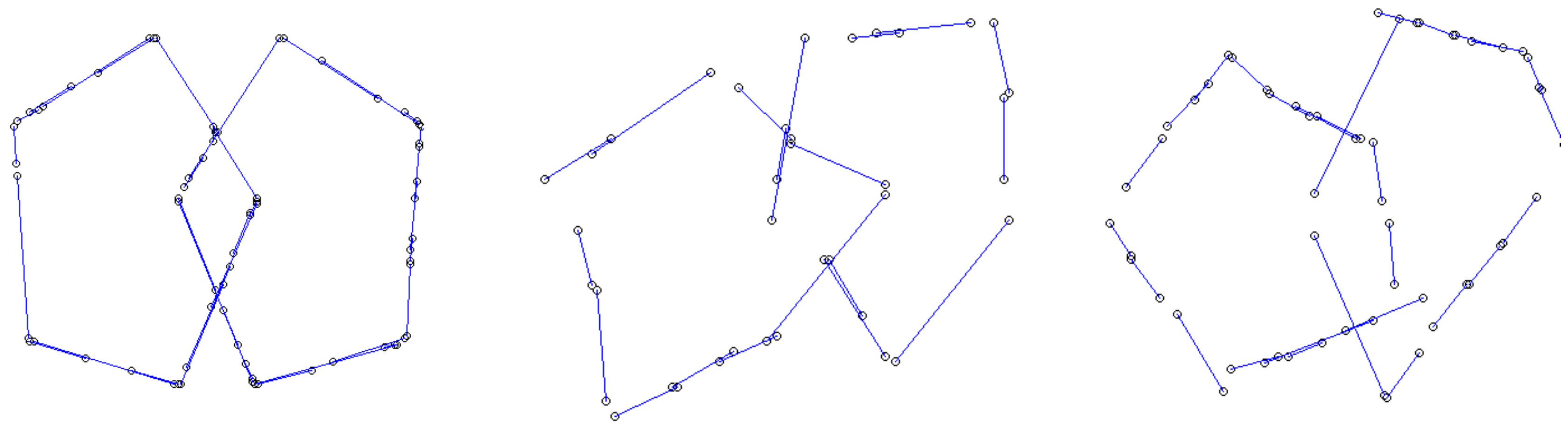

Supplementary Figure 5. Line segments obtained from the Hough transformation. Line segments from the Hough transformation for three PCTs in Supplementary Figure2 were demonstrated.

Supplementary Figure 6

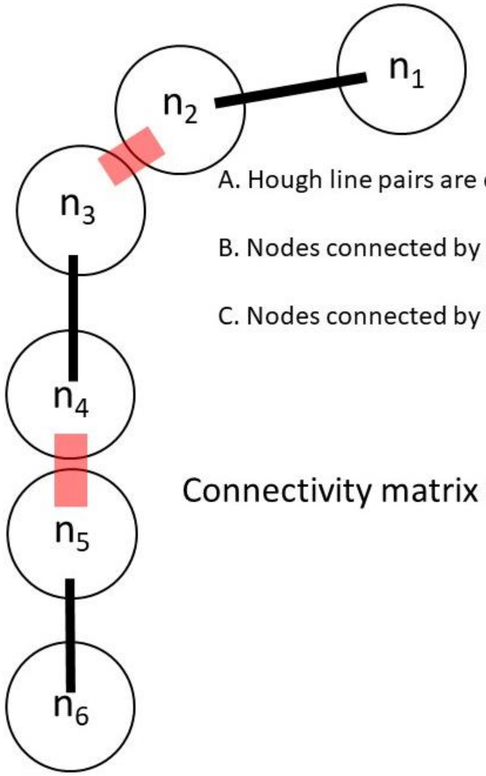

A. Hough line pairs are connected through a solid black line: (n1,n2), (n3,n4), and (n5,n6)

B. Nodes connected by presence of Hough lines: (n3,n5), (n3,n6), and (n4,n6)

C. Nodes connected by a gap within a tolerance (red): (n2,n3), (n4,n5).

Connectivity matrix (C^1) =

node	1	2	3	4	5	6
1	0	1	0	0	0	0
2	1	0	1	0	0	0
3	0	1	0	1	1	1
4	0	0	1	0	1	1
5	0	0	1	1	0	1
6	0	0	1	1	1	0

Supplementary Figure 6. First-order Connectivity matrix. We demonstrated a first-order connectivity matrix consisting of six nodes.

Supplementary Figure 7

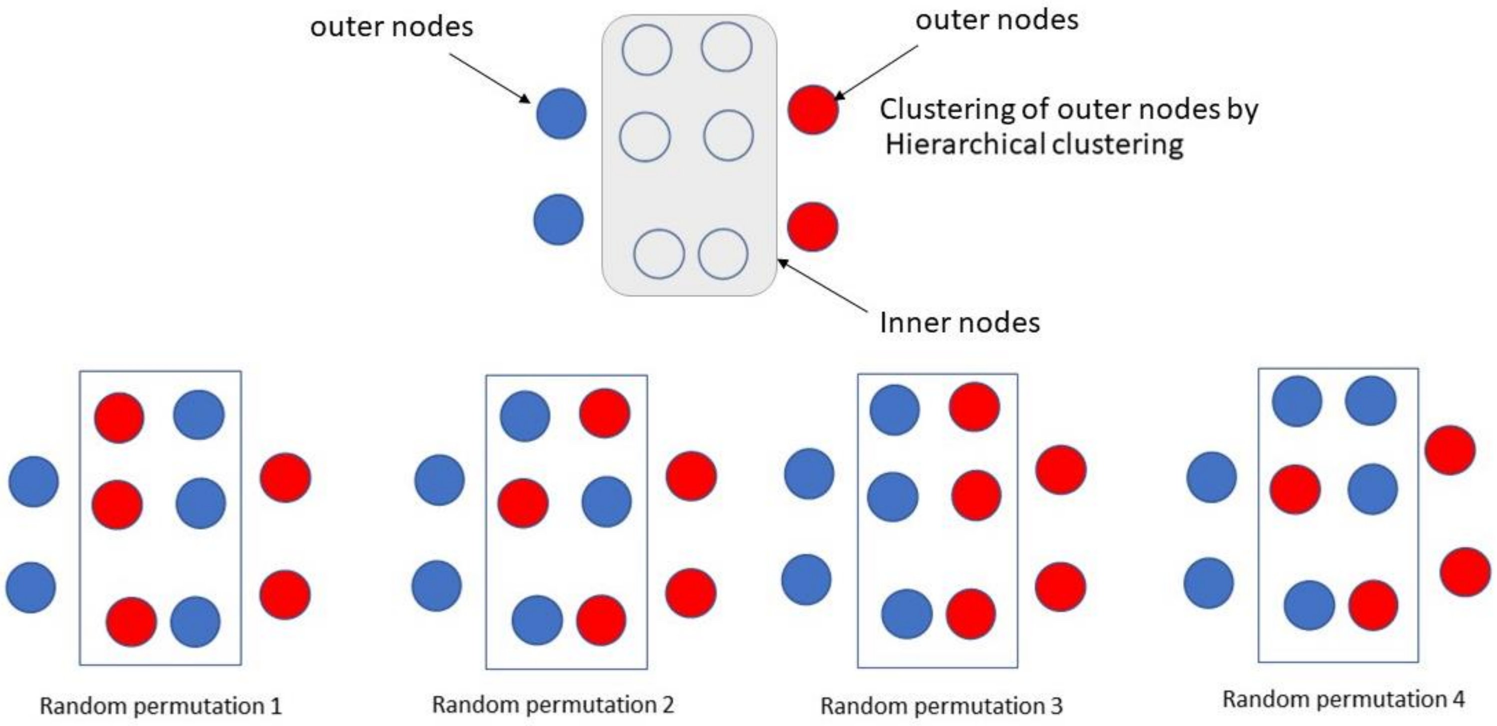

Supplementary Figure 7. Clustering. A demonstration of complete clustering incorporating both outer and inner clustering results was performed. The outer nodes were clustered into their respective pentagons (red, blue). Subsequently, the inner nodes were clustered, and the resulting clusters were combined with the outer node clusters.

Supplementary Figure 8

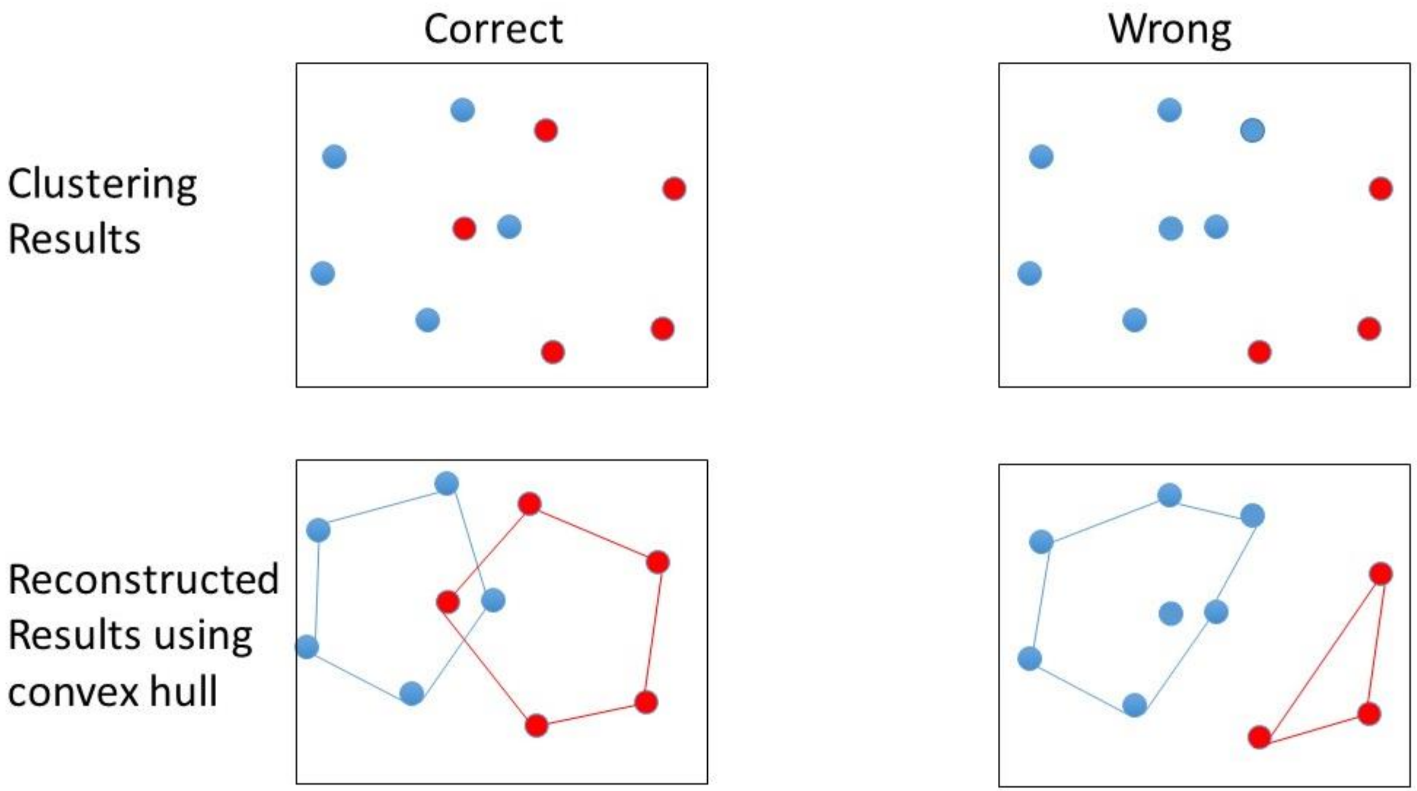

Supplementary Figure 8. Reconstructed pentagons using convex hull after clustering. Demonstrations of the process showing reconstruction of pentagons by applying convex hull with clustering outputs.

Supplementary Figure 9

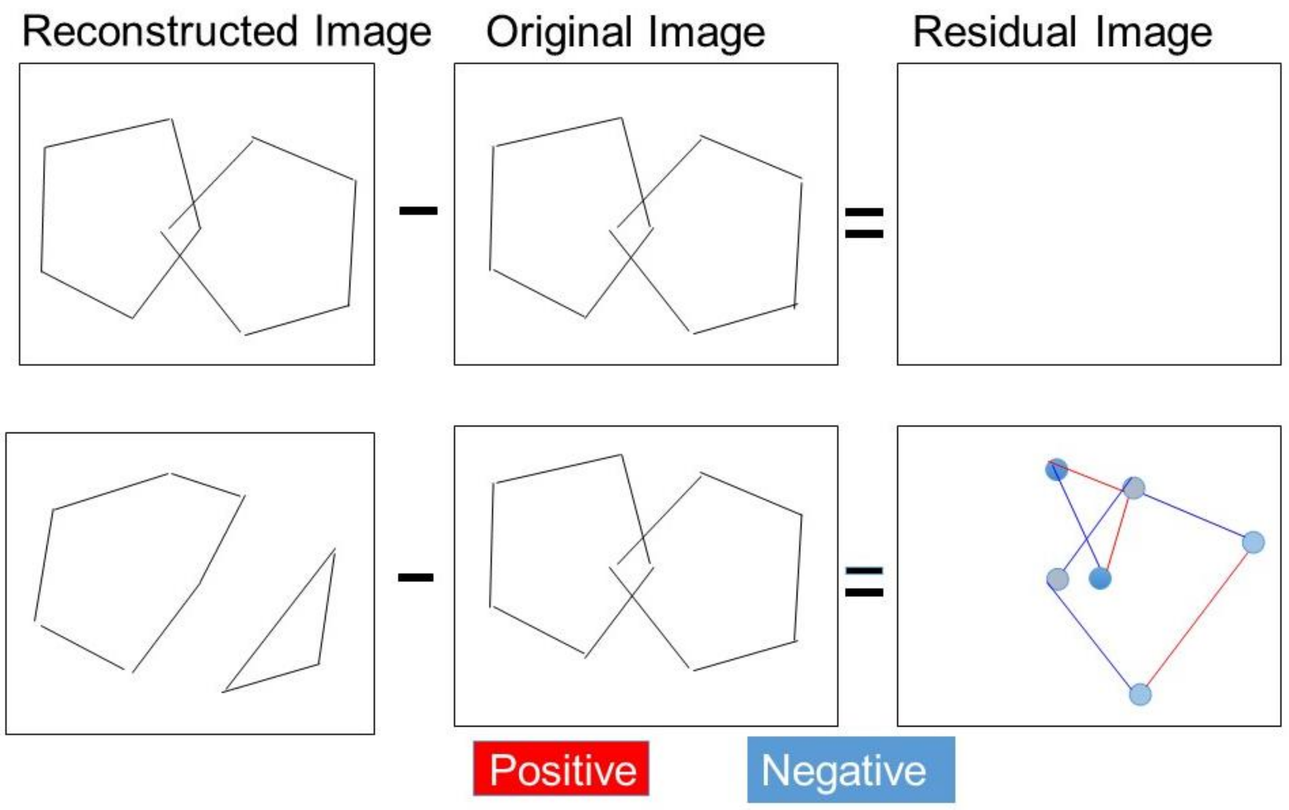

Supplementary Figure 9. Residual images from the reconstructed and the original PCTs. The residual image was generated by subtracting the original PCT image from the reconstructed pentagons shown in Supplementary Figure 8.

Supplementary Figure 10

Original image

Separated pentagons

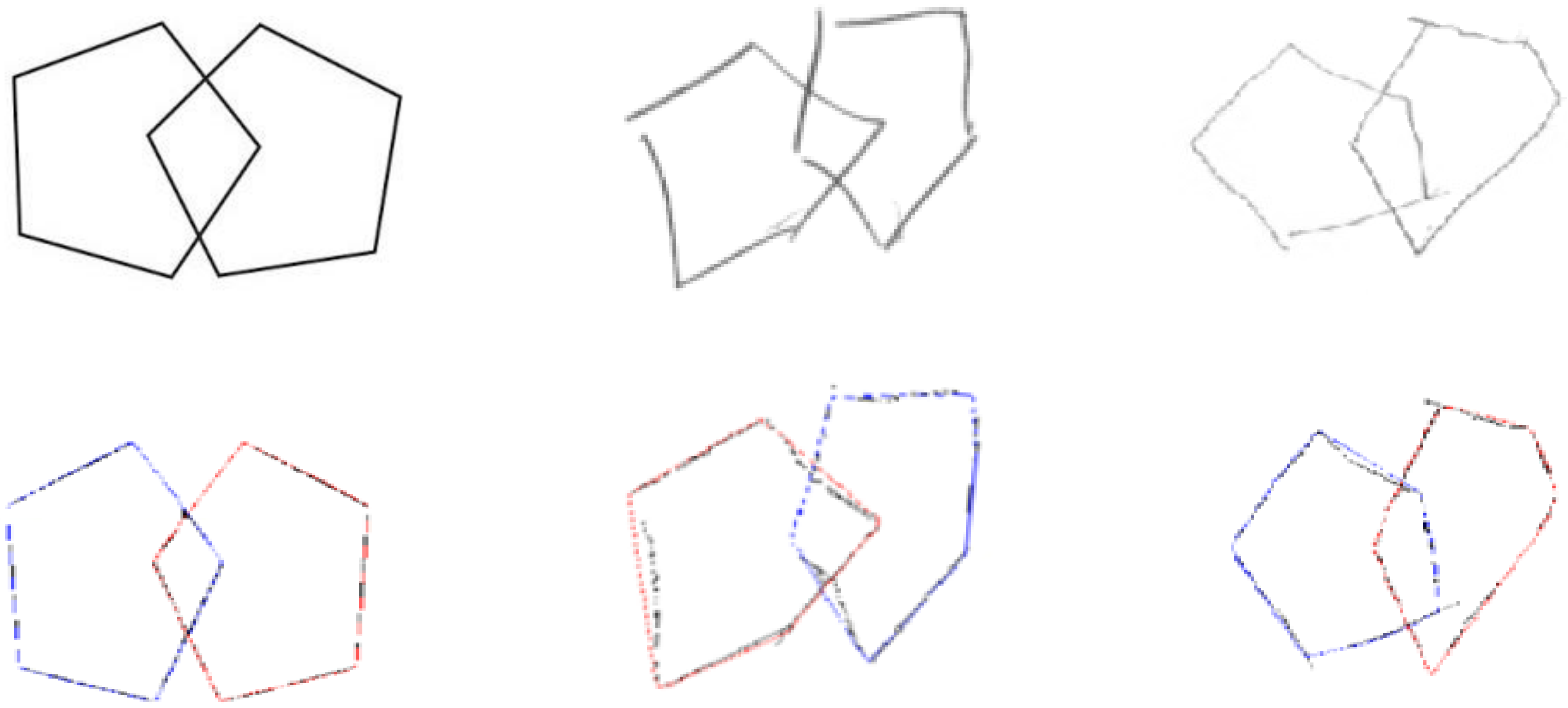

Supplementary Figure 10. The best clustering results for the three demonstrated examples and the reconstructed pentagons using convex hull. The reconstructed pentagons, obtained using the convex hull with the best clustering result, were superimposed on each of the three presented examples.

Supplementary Figure 11

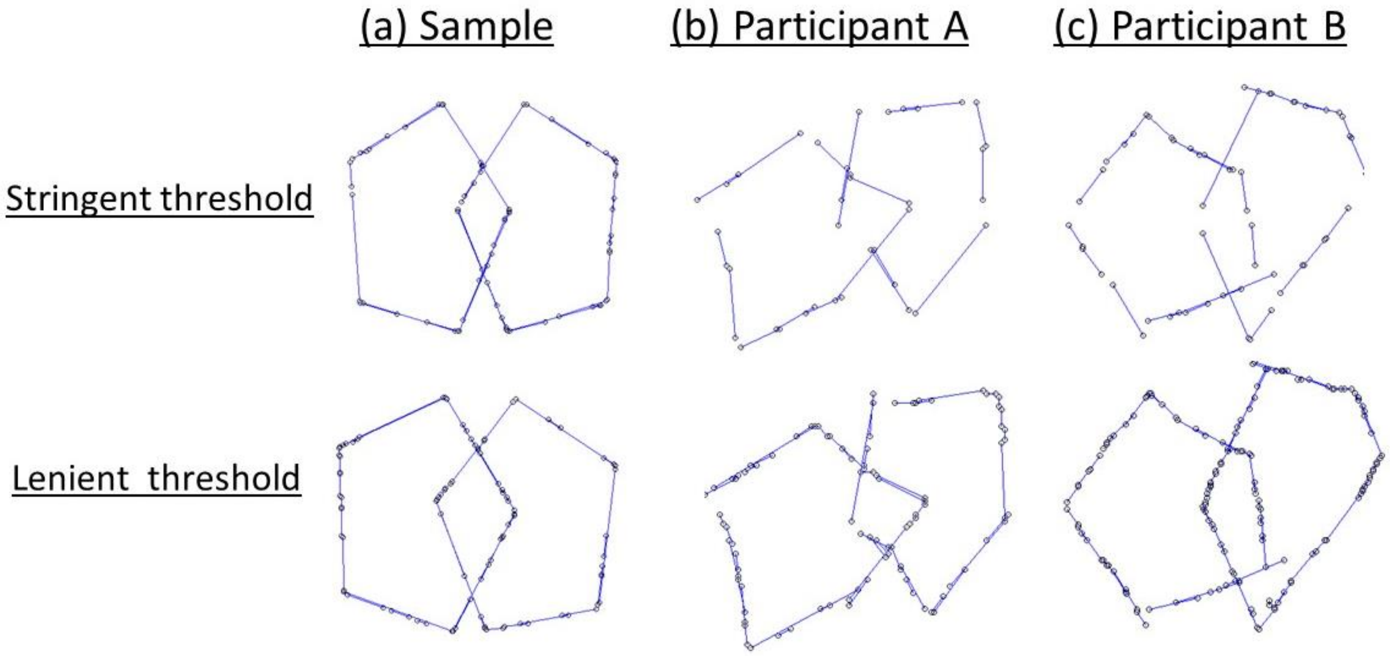

Supplementary Figure 11. Line segments generated with two sets of thresholds from the Hough transformation. We present the differences in line segments using two sets of thresholds: stringent and lenient. The first row displays the line segments obtained using a set of stringent thresholds (10, 10, 4), while the second row shows the line segments obtained using a set of lenient thresholds (1, 2, 4). In these thresholds, the first component represents the minimal number of points sharing the Hough parameters, the second component denotes the minimal length of a line, and the third component indicates the minimal allowed gap between two distinct line segments.
