# Supplementary material for: Digital quantification of the MMSE interlocking pentagon areas: a three-stage algorithm
Source: Sci Rep. 2024 Apr 19;14:9038. doi: 10.1038/s41598-024-59194-1 (PMC11031600; doi:10.1038/s41598-024-59194-1)
Supplement: Supplementary file 2 — Supplementary Information 2. [file 41598_2024_59194_MOESM2_ESM.docx]

Supplementary Material 1. The QIP algorithm

The QIP algorithm comprises three stages: (1) line segment detection from the image, (2) unraveling of two interlocking pentagons, and (3) quantification of the areas of interest. For the purpose of demonstrating the algorithm, we chose three PCTs: the sample interlocking pentagon and two additional PCTs from different participants. Participant A was 79 year old, while Participant B was 92 year old. These three PCTs are illustrated in Supplementary Figure 2.

## **Stage 1. Detection of edges and line segments**

## **1.1 The Canny edge detection**

The Canny edge detection algorithm ^24^ simplifies the image by keeping only boundaries of the input image. The Canny edge detection algorithm detects pixels in digital image that display a sharp change in intensity, often referred to as edges. Output from the Canny edge detection algorithm is a binary image with a positive value for detected edges. Edges contain shape information and thus are the most important features for image recognition and classification. Keeping only edges in image reduces the amount of data to be processed. Using the image containing only edges from the Canny edge detection algorithm, we identified line segments which resulted in a further reduction of image data into a collection of two end points for each line segment. We demonstrated the Canny edge detection with the three example PCTs in Supplementary Figure 3. Details of the Canny edge detection algorithm are shown in Supplementary Material 2A.

## **1.2 The Hough transformation**

In two-dimensional Euclidean space, a line can be parameterized by the y-intercept$(\alpha)$and slope$(\beta)$. Although this parameterization is most extensively adopted, it is not able to represent vertical lines for which the slopes are infinity, *i.e*.,$\beta=\infty$. For this reason, the Hough transformation^25^ is a useful alternative parametrization of lines that enables to detect lines in image using a pair of parameters $(r,\theta)$: the angle$(\theta)$ between a line perpendicular to the line ($l$) and the positive x-axis; the shortest distance $(r)$ from the origin (0,0) to the line $l$. Each image from the Canny edge detection algorithm ^24^ was processed by the Hough transformation ^25^ to identify line segments. The pair of parameters $(r,\theta)$ is referred to as the Hough parameters. That is, a line in the Euclidean space is transformed to a point in the Hough space with (x-axis: *θ*, y-axis: *r*). Any points $(x,y)$ on a line in Euclidean space is thus alternatively represented by a pair of $(r,\theta)$ in the Hough space as follows.

$r=x\cos\theta+y\sin\theta$, where .

Images were reconstructed with line segments identified from the Hough transformation ^25^. To keep line segments with higher authenticity, three thresholds were applied. Those are threshold for (a) minimal number of points sharing the Hough parameters, (b) minimal length of a line, and (c) minimal gap allowed between two distinct line segments. Two Hough lines with a gap less than the minimal gap were merged. Higher the number of points sharing the same Hough parameters and longer length of a line signify more authenticity of a line. We demonstrated the Hough transformation ^25^ with the three example PCTs in Supplementary Figure 4 and the estimated line segments in Supplementary Figure 5. Details of the Hough transformation ^25^ are shown in Supplementary Material 2B.

## **Stage 2. Separation of two interlocking pentagons**

Because two pentagons intersect, quantifying the areas of individual pentagons and their intersection can be complex and challenging. To simplify the computation process, we first aimed to separate the two pentagons. This was done using the line segments acquired in Stage 1, where the line segments were grouped into two individual pentagons. Each line segment detected from the Hough transformation has two endpoints. We call each of the endpoints of a line segment a node so that each line segment has two nodes. It is important to note that assigning the nodes around the centroid, which represents the gravity of all nodes, to their respective pentagons poses a higher level of complexity due to the intersection occurring near the centroid. To address this, we implemented a cut-off distance from the centroid, designating nodes in close proximity as inner nodes, and those further away as outer nodes. We provided further details on cut-off distance in Supplementary Material 2C. To cluster the nodes into their respective pentagons, we employed different approaches. For the outer nodes, we utilized hierarchical clustering, while for the inner nodes, which required more computational intensity due to their proximity to the centroid, we adopted a hybrid approach combining hierarchical clustering and random permutation methods.

## **2.1 Connectivity matrix of nodes from line segments**

A hierarchical clustering algorithm requires distance metric between nodes. To meet the need, we developed a connectivity matrix using nodes from the line segments. We start by defining the first-order connectivity (**C**^1^) among the nodes. It is noteworthy that two nodes of a Hough line segment were trivially connected. We sought connectivity beyond such a trivial connection to have complete connectivity in the method described below. Specifically, we draw an extended line ($\bar{e}_{ij}$) connecting arbitrary two nodes (*i*) and (*j*), and examine presence of any line segments that are found between two designated nodes or being in a proximity to $\bar{e}_{ij}$within tolerance levels based on angle and distance. In addition, we allowed gap between two nodes that any two nodes within a tolerance were marked as connected. Allowance of gap was necessary for enhanced connectivity between two nodes particularly located around corners because angles between the extended line ($\bar{e}_{ij}$) and nearby line segments were beyond a tolerance. In addition, we defined connectivity of a node to itself as zero. The first-order connectivity matrix is a square and symmetric matrix, which is an adjacency matrix with undirected edges in graph theory ^28^. The first-order connectivity was defined in Eq. (1). Details of construction of the first-order connectivity is presented in Supplementary Figure 6. The first-order connectivity matrix was then transformed into higher-order connectivity. Specifically, the second-order connectivity was defined as the result of multiplying the first-order connectivity matrix by itself, indicating the connectivity between two nodes through an intermediate node. Similarly, the *m*-th order connectivity (m > 1) was defined as the result of multiplying the first-order connectivity matrix m times, as shown in Eq. (2). We defined a total connectivity using connectivity matrix up to *m*-th order where *m* was set empirically to give entire connectivity of the nodes considered (*m* ≤5) in Eq. (3). The connectivity matrix was subsequently converted into a distance metric by taking the inverse of the exponentiated connectivity matrix, as described in Eq. (4).

$\begin{matrix} \mathbf{C}^{1}(i,j;i\neq j)= \left\{\begin{matrix} 1, \text{if} \text{line segements exsit between} n_{i}\text{ and }n_{j} \text{or} \text{gap} (n_{i},n_{j})< \text{tol,} \\ 0, \text{otherwise} \text{ } \text{ } \end{matrix} \right. \\ \text{and } \\ \mathbf{C}^{1}(i,i) = 0. \end{matrix}$(1)

$\boldsymbol{C}^{m}=\overset{m-times}{\overset{⏠}{\boldsymbol{C}^{1}\times\cdots\times\boldsymbol{C}^{1}}}$ (2)

$\mathbf{TC}=\sum_{i=1}^{m} \mathbf{C}^{i}$ (3)

$\mathbf{D}(i,j)=\exp\left[-\mathbf{TC}(i,j)\right]$ (4)

## **2.2 Clustering of line segments to each pentagon**

Using the distance matrix **D** in Eq. (4), we initiated clustering with the outer nodes. For the outer nodes, we applied a hierarchical clustering method using Wald's minimum within-cluster variance criterion ^29^ and the distance metric described in Eq. (4). Additional details regarding the hierarchical clustering procedure can be found in Supplementary Material 2D. Following this step, each node was assigned a label indicating whether it belongs to pentagon 1 or pentagon 2.

To assign the inner nodes to their respective pentagons, we employed a random permutation approach in conjunction with hierarchical clustering. The inner nodes were initially grouped together based on their connectivity according to the distance matrix using hierarchical clustering. Subsequently, the classification of inner clusters into individual pentagons was achieved by randomly permuting the class labels (1 or 2) assigned to each inner cluster. The total number of random permutations, considering the number of inner clusters, increased exponentially. Each clustering result obtained with the inner nodes was paired with the clustering results obtained with the outer nodes to create a comprehensive clustering outcome. A diagram illustrating the combined clustering of outer and inner nodes is presented in Supplementary Figure 7.

Total number of permutation = 2^#{IC}^,

where #{IC} is the number of inner clusters. The total number of whole clustering results therefore is as much as the total number of permutations with the number of inner clusters.

## **2.3 Determination of the best clustering outcome**

Considering that a pentagon is a convex polygon, we aimed to reconstruct the entire PCT by combining two reconstructed individual pentagons. Each pentagon was reconstructed by applying a convex hull with the set of nodes assigned. We expected that the reconstructed PCT would exhibit minimal deviation from the original PCT if clustering of nodes to respective pentagon was done correctly. Therefore, the best clustering result was determined based on the minimum difference between the original image and the reconstructed image. To achieve this, we reconstructed a PCT by applying a convex hull to each of the comprehensive clustering results. The best clustering result was determined by evaluating the mean square error (MSE) of the residual image, which represents the deviation between the reconstructed PCT and the original image. Denoting the original image and the reconstructed image as $\text{I}_{o} \text{and} \text{I}_{r}$, respectively, MSE was calculated over a rectangle Ω that covers both images, as defined in Eq. (5). A diagram illustrating this selection procedure was provided in Supplementary Figures 8 and 9. The best clustering result with a convex hull applied for each pentagon overlaid on original image is demonstrated in Figure 10.

$\text{MSE}=\frac{1}{\text{AREA}(\Omega)}\sum_{(x,y)\in\Omega} (\text{I}_{o}(x,y)-\text{I}_{r}(x,y))^{2}$ , (5)

## **Stage 3. Quantification of areas of interest**

We quantify each area of pentagon using a built-in function of MATLAB (name: polyarea), which counts the number of pixels in a 2D polygon image. We quantify the areas of intersection using the Monte Carlo Integration ^26^, where we randomly draw samples (*x, y*) from a rectangle covering the intersecting area, and count the random samples falling inside the area of intersection as in Eq. (6). We denote the region of intersection of two pentagons as I_12_.

$\text{AREA}(\text{I}_{12})=\text{AREA}(\Omega)\times\frac{1}{L}\sum_{i=1}^{L} f_{12}(x_{i},y_{i})$, (6)

where (*x_i_,y_i_*), *i* = 1,2,…, *L*, are random samples selected from the inside of a rectangle Ω that covers the entire PCT images I_1_ and I_2_., The function *f*_12_ is defined as

$f_{12}\left(x_{\mathbf{i}},y_{\mathbf{i}}\right)= \left\{\begin{matrix} 1, \text{if} (x_{\mathbf{i}},y_{\mathbf{i}}) \text{is} \text{located inside} \text{the} \text{intersection} \text{area} \\ 0, \text{otherwise} \text{ } \end{matrix} \right.\text{ }$.

Using the measures of the areas of individual pentagons and their intersection, we quantify the total area of two interlocking pentagon, the proportion of the intersection as,

$\text{total AREA}=\text{AREA}(\text{I}_{1})+\text{AREA}(\text{I}_{2})-\text{AREA}(\text{I}_{12})$,

$0\leq\text{Proportion of Intersection}=\frac{\text{AREA}(\text{I}_{12})}{\text{total AREA}}\leq1$ ,

and a balance ratio of the two pentagon areas in terms of smaller area to larger area as follows.

$0\leq\text{Balance} \text{ratio}=\frac{\min(\text{AREA}(\text{I}_{1}), \text{AREA}(\text{I}_{2}))}{\max(\text{AREA}(\text{I}_{1}), \text{AREA}(\text{I}_{2}))}\leq1$ .

## **Computation time and software**

We measured the computation time using the sample pentagon, running on a 64-bit Linux system with a quad-core processor (with two threads per core) and 126 GB of random access memory. The computation time for the sample interlocking pentagon was 73 seconds. However, it's worth noting that cases requiring manual corrections or exploration of non-default parameters set for the QIP which depends on the shapes of individual PCT may take longer time to process. We adopted Python, R, and MATLAB at different stages and functions of the QIP algorithm.

Supplementary Material 2. Detailed Components of the QIP algorithm

1. **The Canny edge detection**

The Canny edge detection algorithm ^24^ simplifies the image by keeping only boundaries of the input image. Output from the Canny edge detection algorithm is a binary image with a positive value for detected edges. The Canny edge detection algorithm consists of five steps: (a) applying of a Gaussian filter to smooth the image; (b) assessing intensity gradient of the image using the Sobel filter; (c) suppressing non-maximum pixels to remove spurious response and thus make edges thinner; (d) applying double threshold to determine potential edges; and (e) edge tracking by hysteresis suppressing of all other edges that are weak or not connected to strong edges.

1. **The Hough transformation**

Images only with edges identified by the Canny edge detection algorithm ^24^ were transformed into the Hough space. Images were reconstructed with line segments identified from the Hough transformation ^25^. To keep line segments with higher authenticity, three generally adopted thresholds were applied. Those are threshold ($\gamma_{1}$) for minimal number of points sharing the Hough parameters, a minimal length of a line ($\gamma_{2}$), and a minimal gap allowed between two distinct line segments ($\gamma_{3}$). Two Hough lines with a gap less than $\gamma_{3}$ were merged. More stringent thresholds remain only line segments with higher authenticity and longer length. However, applying higher thresholds could lose line segments that do not meet the thresholds such as short lines. We demonstrate such effects using two sets of thresholds, striengent vs. lenient, of $(\gamma_{1},\gamma_{2},\gamma_{3})$. In Supplementary Figure 11, the first row shows the reconstructed images using a set of stringent thresholds (10, 10, 4), and the second row using a set of lenient thresholds (1, 2, 4). As results, reconstructed imges on the first row in the figure showed smaller number of line segments and longer length of the Hough lines with (10, 10, 4) than with (1, 2, 4). Furthermore, gaps between the Hough lines were larger with (10, 10, 4) because the lines that didn’t meet the minimal number threshold ($\gamma_{1}$) and minimal length ($\gamma_{2}$) were discarded.

1. **Designating nodes to inner and outer nodes**

To distinguish between inner nodes and outer nodes, we introduced a cut-off distance from the centroid. Inner nodes were defined as nodes in close proximity to the centroid, while outer nodes were those located further away. To ensure optimal performance and avoid excessive processing time, it was crucial to select the appropriate set of inner nodes. To determine the set of inner nodes, we sorted the nodes based on their distance from the centroid in ascending order. Initially, we included nodes up to the 40th percentile as inner nodes. However, the cut-off distance could be adjusted based on the accuracy of clustering with the outer nodes. A higher cut-off distance might be necessary in cases where the quality of clustering with the outer nodes is compromised.

1. **Hierarchical Clustering algorithm**

Hierarchical clustering, employing Wald's minimum within-cluster variance criterion^29^, is an agglomerative approach that constructs clusters by merging existing clusters at each step of the hierarchy. The objective is to minimize the within-cluster variance when determining which clusters to merge. Initially, each observation is considered as its own cluster at the first step and then grow combining other nodes closely connected.
